# Effects of Heart Rate-Synchronized Breathing on Anaerobic Threshold During Incremental Exercise Testing: A Preliminary Case Series

**DOI:** 10.7759/cureus.114027

**Published:** 2026-08-05

**Authors:** Dai Kawakita

**Affiliations:** 1 Faculty of Health and Medical Sciences, Department of Physical Therapy, Teikyo Heisei University, Tokyo, JPN

**Keywords:** anaerobic threshold (at), cardiopulmonary exercise testing (cpx), exercise tolerance, heartbeat-synchronized breathing, respiratory sinus arrhythmia (rsa)

## Abstract

Heart rate-synchronized breathing (HRSB) -- a technique in which the respiratory cycle is entrained to the cardiac cycle -- may optimize autonomic nervous function and ventilatory efficiency, potentially improving exercise tolerance. However, its effect on the anaerobic threshold (AT) remains unclear. This study examined the influence of HRSB on AT and related physiological indices during incremental cycle ergometry in healthy young adults.

Three healthy male adults (mean age: 22.3 ± 1.2 years) underwent two cardiopulmonary exercise tests on separate days: Day 1 (normal breathing, control) and Day 2 (HRSB intervention). During HRSB, participants inhaled for 2 heartbeats and exhaled for 4 heartbeats (2:4 ratio) guided by auditory cardiac-cycle cues. Respiratory-cardiac entrainment rate, AT oxygen uptake (AT-VO₂), ventilatory equivalent for oxygen (VE/VO₂), and subjective rating of perceived exertion (RPE) were compared within individuals.

Participants A and B demonstrated improvements in AT-VO₂ (+2.0 and +1.3 mL/min/kg, respectively), reductions in VE/VO₂ (25.0 to 22.1 and 24.3 to 21.8), and lower RPE scores (13 to 11 and 13 to 12). Participant C showed a marked decline in AT-VO₂ (−4.9 mL/min/kg) and a substantial increase in VE/VO₂ (16.0 to 25.7), indicating deteriorated ventilatory efficiency.

HRSB may improve exercise tolerance and ventilatory efficiency in some individuals, likely through enhanced parasympathetic activity and baroreflex sensitivity. However, outcomes varied markedly between participants, suggesting that individual differences in autonomic response and respiratory control capacity significantly moderate the effect. Future studies with larger samples and individualized resonance-frequency assessment are warranted.

## Introduction

Respiratory regulation during exercise is a critical factor influencing exercise efficiency and ratings of perceived exertion, and numerous studies have investigated the synchronization between exercise rhythm and respiration or heart rate [1,2]. Locomotor-respiratory coupling (LRC) -- the entrainment of the breathing cycle to the movement cycle -- is a phenomenon widely observed in bipedal and quadrupedal animals. Bramble and Carrier demonstrated that humans and other mammals tend to synchronize respiratory and stride rhythms at integer ratios (e.g., 1:1 or 2:1) during running [3]. Bernasconi and Kohl analyzed the incidence and patterns of coordination between breathing and leg movement rhythms during running, and demonstrated that intentional pacing of breathing to the exercise rhythm significantly increased the degree of locomotor-respiratory coordination [4]. Garlando et al. additionally demonstrated that coupling the breathing and cycling rhythms significantly reduces oxygen uptake at equivalent workloads [5]. These foundational observations have been extended by more recent research. Harbour et al. conducted a comprehensive synthesis of evidence-based breathing strategies for running, concluding that LRC and slow breathing techniques may reduce exercise-induced dyspnea and improve running tolerance, although the optimal strategy depends on individual characteristics and exercise intensity [6]. More recently, Harbour et al. demonstrated in novice female runners that step-adaptive sound guidance significantly enhances LRC frequency coupling during running, highlighting the potential of externally paced breathing interventions to optimize cardiorespiratory synchronization during exercise [7]. Nevertheless, the extent to which cardiac-cycle-entrained breathing specifically contributes to exercise tolerance and the anaerobic threshold (AT) has not been fully elucidated.

Regarding the relationship between respiration and heart rate, respiratory sinus arrhythmia (RSA) -- the phenomenon whereby heart rate increases during inspiration and decreases during expiration -- has long attracted research attention. RSA is widely used as an index of autonomic nervous regulation mediated by the vagus nerve [8]. Lehrer et al. demonstrated that heart rate variability (HRV) biofeedback involving slow-paced breathing at an individual's cardiovascular resonance frequency increases vagal baroreflex gain and improves peak expiratory flow [9]. The mechanism by which respiratory patterns influence circulatory dynamics through HRV has thus become a focus of research. In addition, breathing with a prolonged expiratory phase (inspiratory-to-expiratory ratio of 1:2) has been reported to augment parasympathetic nervous activity, reduce the rate pressure product, and improve ventilatory efficiency during incremental exercise [10]. Prolonged expiratory breathing has also been shown to promote parasympathetic dominance as assessed by HRV at rest [11,12], suggesting broader autonomic benefits that may contribute to improved exercise efficiency.

The AT is the inflection point at which lactate production increases sharply with rising exercise intensity and is a representative index for evaluating endurance exercise capacity. AT is widely used in exercise prescription and rehabilitation settings, and elevating the AT implies an improvement in exercise tolerance. Wasserman et al. explained the physiological and biochemical basis of the AT, describing how accelerated glycolysis above the AT produces lactic acidosis that is buffered by bicarbonate, resulting in a steeper increase in CO₂ output detectable by gas exchange [13]. The V-slope method, established by Beaver et al., provides a standard gas-exchange-based approach to non-invasive AT determination [14]. Matsumoto et al. demonstrated that prolonged expiratory breathing augments parasympathetic nervous activity, improves ventilatory efficiency, and increases oxygen uptake during incremental exercise [10]. Prolonged expiratory breathing has also been shown to promote parasympathetic dominance as assessed by HRV at rest [11,12], making the potential impact of such breathing interventions on AT a clinically important issue.

The present study focused on "heart rate-synchronized breathing" (HRSB), a breathing intervention in which the respiratory rhythm is entrained to the cardiac cycle, and aimed to examine its effects on AT. Drawing on prior research demonstrating that prolonged expiratory breathing augments parasympathetic nervous activity and improves ventilatory efficiency during incremental exercise [10], and that slow paced breathing at an individual's resonance frequency enhances baroreflex sensitivity [9], we sought to verify the effects of HRSB on ventilatory response, autonomic nervous activity, and exercise efficiency (VO₂/W ratio) during an incremental exercise test, and to quantify changes in AT.

## Case presentation

Clinical presentation and participant characteristics

Three healthy adult males in their twenties (mean age: 22.3 ± 1.2 years; Participants A, B, and C) were enrolled. All participants were physically active university students enrolled at Teikyo Heisei University with no history of respiratory, cardiovascular, or orthopedic conditions requiring exercise restriction, and none were receiving medication at the time of enrollment. The sample was limited to three participants due to the preliminary and exploratory nature of the study, which aimed to generate initial within-individual data to inform the design of future larger-scale investigations. Participants were selected consecutively from among eligible volunteers who met the inclusion criteria and were available during the study period; no formal randomization was applied. Baseline cardiorespiratory parameters were within normal limits for healthy young males. All participants provided written informed consent before participation, and the study was conducted with approval from the Ethics Committee of Teikyo Heisei University (Approval No.: 2025-058).

Diagnostic workup

Each participant underwent two separate cardiopulmonary exercise tests (CPX) on a cycle ergometer (Ergoselect 200; Inter Rehab Co., Ltd., Japan) to evaluate exercise capacity under two breathing conditions. Tests were conducted in an environment maintained at 22-24°C and 40%-60% relative humidity and were performed at the same time of day for each participant. Participants were instructed to refrain from strenuous exercise, caffeine, and large meals for at least three hours before each test. Expired gas metabolism was measured using a respiratory gas metabolic monitor (Cpex-1; Inter Rehab Co., Ltd., Japan). Electrocardiography (ECG, 3-lead), surface electromyography of the quadriceps femoris, and respiratory cycle signals were recorded simultaneously using PowerLab with LabChart software (Bio Research Center Co., Ltd., Japan). The respiratory phase was detected via a respiratory belt transducer. The AT was determined using the V-slope method applied to expired gas data, as described by Beaver et al. [14] and Wasserman et al. [13]. Baseline measurements of blood pressure, heart rate, respiratory rate, and subjective rating of perceived exertion (RPE) [15] were obtained before and after each test.

**Figure 1:**
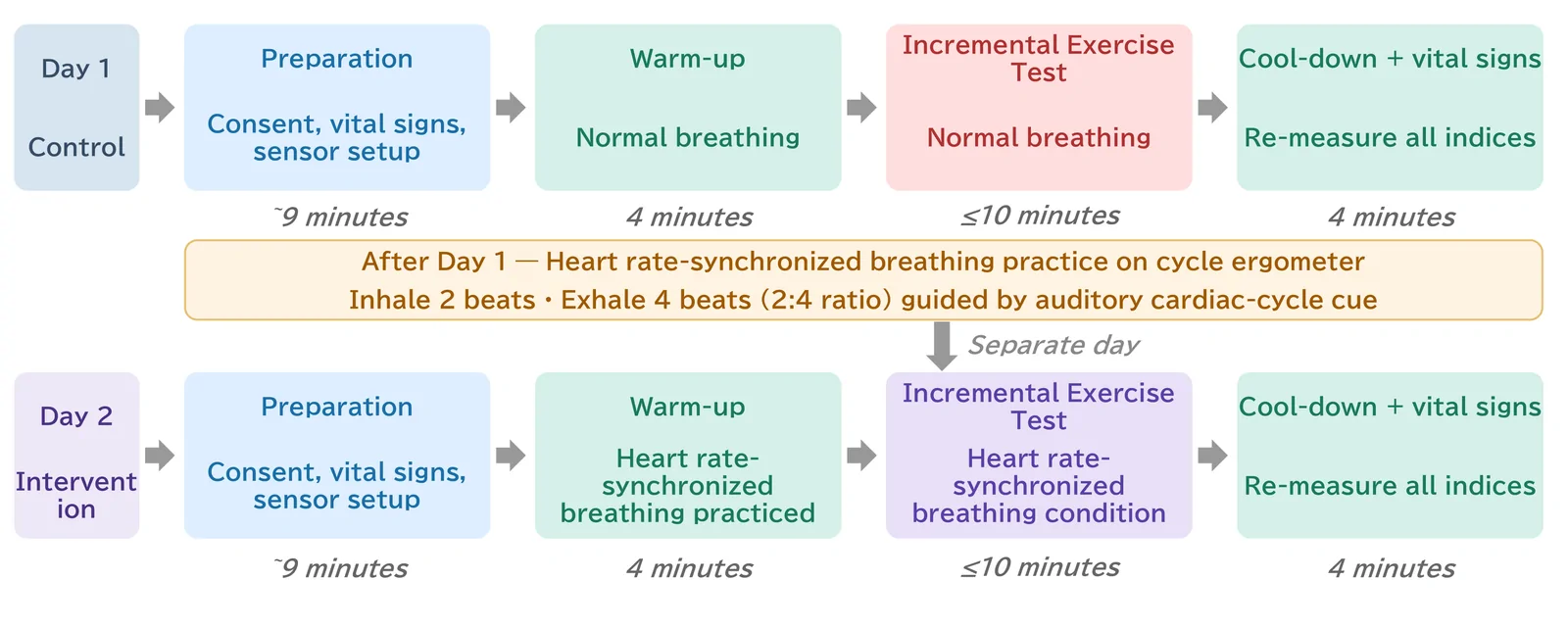
Two-day cardiopulmonary exercise testing protocol Day 1: Incremental cycle ergometer test under normal breathing (control condition). Following completion of Day 1, participants practiced the heart rate-synchronized breathing (HRSB) technique on the ergometer. Day 2: identical incremental exercise test performed under the HRSB condition, with a 2:4 inspiratory-to-expiratory ratio paced by auditory R-wave cues from the electrocardiogram monitor. Both days included a warm-up phase (10 watts, 4 minutes), an incremental loading phase (10 watts per minute until volitional fatigue), and a cool-down phase (10 watts, 4 minutes). Before and after each test, blood pressure, heart rate, respiratory rate, and the Borg rating of perceived exertion were measured. Electrocardiography, electromyography, and expired gas analysis were recorded continuously throughout each test. An automated external defibrillator was available in the room throughout testing. This figure was originally created by the authors using Microsoft PowerPoint (Microsoft Corporation, Redmond, WA, USA).

The exercise protocol consisted of a 4-minute warm-up at 10 W, followed by incremental load increases of 10 W per minute until volitional fatigue, and a 4-minute cool-down at 10 W. Day 1 was performed under normal (spontaneous) breathing as a control condition. On Day 2, participants performed the identical protocol under the HRSB intervention, as illustrated in Figure 1.

Treatment: HRSB intervention

The HRSB intervention involved entraining the respiratory rhythm to the cardiac cycle using a 2:4 inspiratory-to-expiratory ratio -- inhaling for 2 heartbeats and exhaling for 4 heartbeats -- with the audible R-wave signal of the ECG monitor serving as the pacing reference. This ratio was selected based on evidence that a prolonged expiratory phase augments parasympathetic nervous activity and improves ventilatory efficiency during incremental exercise [10], and promotes parasympathetic dominance as assessed by HRV at rest [11]. Standardized verbal instructions were provided from the onset of the warm-up phase to facilitate acquisition of the synchronization pattern. Between Day 1 and Day 2, all participants practiced the HRSB technique on the cycle ergometer to ensure familiarization before the intervention test. HRSB entrainment was defined as a respiratory cycle containing exactly 6 R-waves (i.e., 6 heartbeats per breath), and the entrainment rate was calculated as the proportion of breaths meeting this criterion relative to total breaths.

Clinical course and outcome

**Figure 2:**
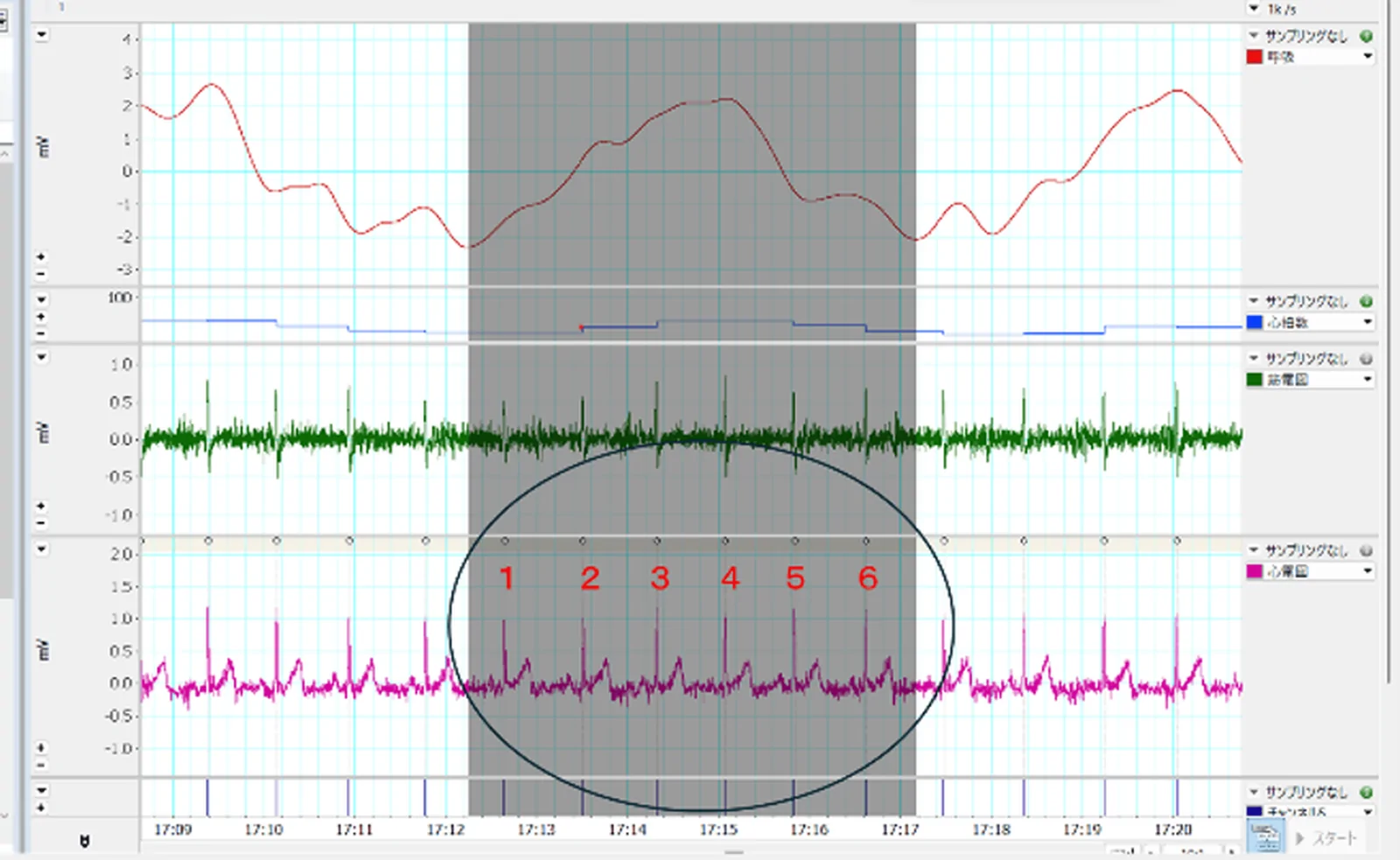
Representative waveform of a synchronized breath during the heart rate-synchronized breathing condition Upper trace: respiratory belt signal; middle trace: surface electromyogram of the quadriceps femoris (vastus lateralis); lower trace: electrocardiogram. The oval highlight demarcates one complete respiratory cycle (from onset of inspiration to the next onset of inspiration) and indicates the heart rate -- that is, the number of R-waves -- counted within that cycle. In this example, exactly 6 R-waves are contained within the highlighted respiratory cycle, fulfilling the entrainment criterion of 6 heartbeats per breath. The regular correspondence between R-wave timing and respiratory phase transitions confirms successful cardiac-respiratory synchronization. Waveforms were originally recorded and exported by the authors using LabChart software (Bio Research Center Co., Ltd., Japan) with a respiratory belt transducer for respiratory phase detection.

**Figure 3:**
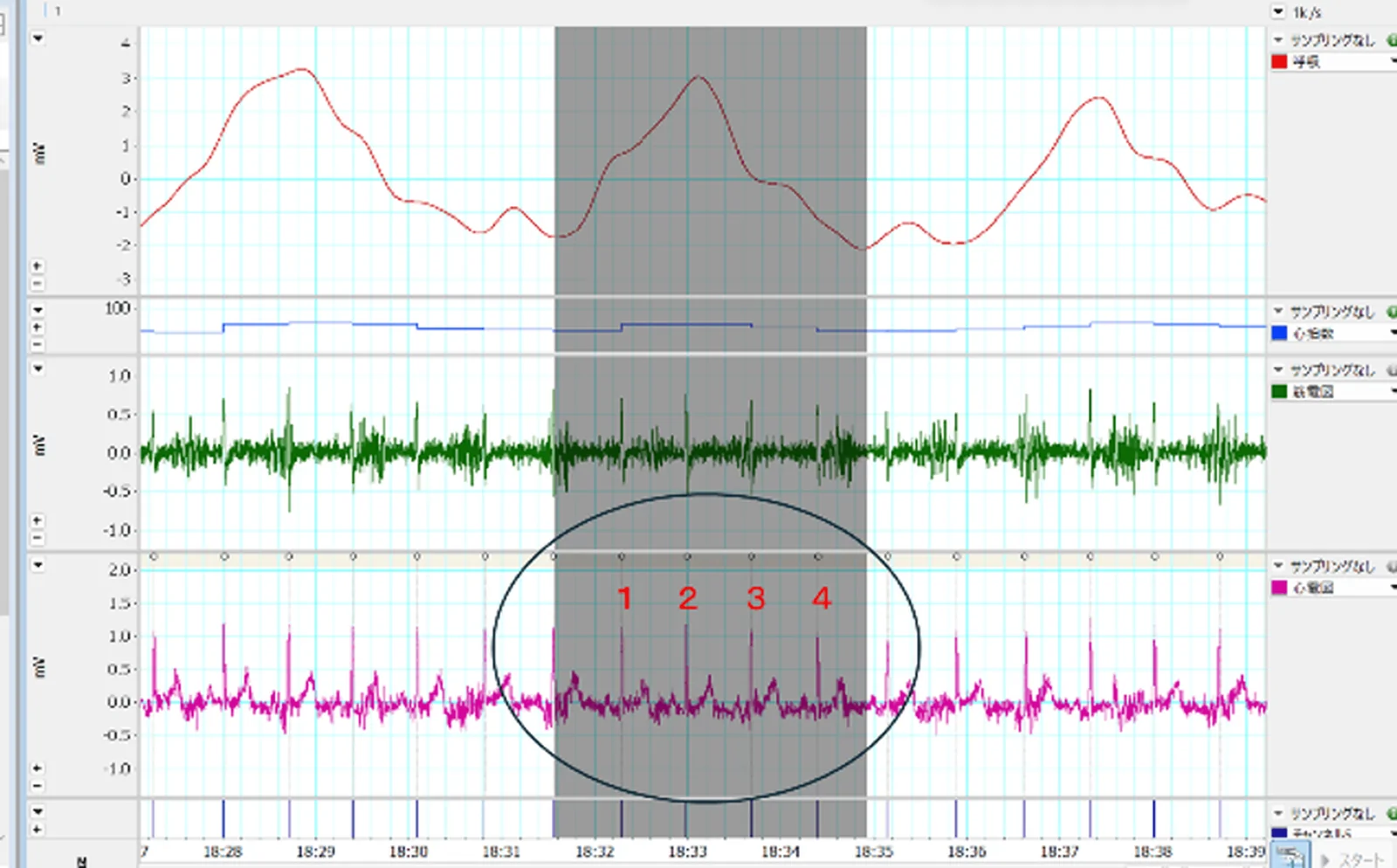
Representative waveform of a non-synchronized breath during the heart rate-synchronized breathing condition Upper trace: respiratory belt signal; middle trace: surface electromyogram of the quadriceps femoris (vastus lateralis); lower trace: electrocardiogram. The oval highlight demarcates one complete respiratory cycle (from onset of inspiration to the next onset of inspiration) and indicates the heart rate -- that is, the number of R-waves -- counted within that cycle. In this example, the number of R-waves within the highlighted respiratory cycle does not fulfill the criterion of exactly 6, and irregular correspondence between respiratory phase transitions and R-wave timing is evident, indicating a loss of cardiac-respiratory synchronization. This breath was therefore classified as non-synchronized and excluded from the entrainment rate calculation. Waveforms were originally recorded and exported by the authors using LabChart software (Bio Research Center Co., Ltd., Japan) with a respiratory belt transducer for respiratory phase detection.

All three participants completed both CPX sessions without adverse events. The entrainment rate on Day 2 exceeded that on Day 1 in all participants, confirming that the HRSB intervention produced a meaningful change in breathing pattern relative to the control condition. Physiological outcomes are summarized in Table 1, and representative synchronized and non-synchronized waveforms are shown in Figures 2, 3, respectively.

**Table 1:** Within-individual changes in physiological indices between the control (Day 1) and heart rate-synchronized breathing (Day 2) conditions [13] AT-VO₂: oxygen uptake at anaerobic threshold; VE/VO₂: ventilatory equivalent for oxygen; RPE: rating of perceived exertion (Borg scale).

Variable	A (Day 1)	A (Day 2)	B (Day 1)	B (Day 2)	C (Day 1)	C (Day 2)
AT-VO₂ (mL/min/kg)	24.2	26.2	22.8	24.1	26.5	21.6
VE/VO₂ at AT	25	22.1	24.3	21.8	16	25.7
Borg RPE at AT	13	11	13	12	11	15
Entrainment rate (%)	15.3	48.6	11	46.9	7	48

Participant A (AT-VO₂ at baseline: 24.2 mL/min/kg) demonstrated a clinically meaningful improvement under the HRSB condition, with AT-VO₂ increasing to 26.2 mL/min/kg (+2.0 mL/min/kg). Ventilatory efficiency improved, as reflected by a reduction in VE/VO₂ from 25.0 to 22.1, and subjective exertion decreased from an RPE of 13 to 11. Exercise duration and workload at AT were both higher under the HRSB condition.

Participant B (AT-VO₂ at baseline: 22.8 mL/min/kg) showed a similar pattern of improvement, with AT-VO₂ increasing to 24.1 mL/min/kg (+1.3 mL/min/kg), VE/VO₂ decreasing from 24.3 to 21.8, and RPE declining from 13 to 12, consistent with enhanced ventilatory efficiency and reduced subjective exercise burden.

Participant C (AT-VO₂ at baseline: 26.5 mL/min/kg) showed a contrasting response. Under the HRSB condition, AT-VO₂ decreased markedly to 21.6 mL/min/kg (−4.9 mL/min/kg), VE/VO₂ increased substantially from 16.0 to 25.7, and RPE increased from 11 to 15, indicating a deterioration in ventilatory efficiency and an increase in subjective exercise burden. This participant's entrainment rate was 48%, placing him in the intermediate range among the three participants. A representative example of a synchronized breath is shown in Figure 2, and a non-synchronized breath is shown in Figure 3.

## Discussion

This preliminary case series examined physiological responses to HRSB in three healthy young adults undergoing incremental cycle ergometry. The principal finding is that the effect of HRSB on AT was markedly heterogeneous: Participants A and B demonstrated improvements in AT-VO₂, VE/VO₂, and RPE, whereas Participant C showed deterioration across all three indices. This polarization of outcomes underscores the importance of individual differences in modulating the physiological response to HRSB.

In Participants A and B, increases in AT-VO₂ were accompanied by reductions in VE/VO₂ and RPE, consistent with improved ventilatory efficiency and attenuated sympathetic activation during submaximal exercise. The 2:4 inspiratory-to-expiratory ratio employed in HRSB is aligned with prior evidence that prolonged expiratory breathing augments parasympathetic nervous activity, reduces rate pressure product, and improves ventilatory efficiency during incremental exercise [10]. By stabilizing the respiratory rhythm, HRSB may have reduced the ventilatory cost per unit of oxygen uptake, thereby elevating the AT-VO₂. This interpretation is further supported by Garlando et al., who reported that synchronization of breathing with cycling rhythm significantly reduced oxygen uptake at equivalent workloads [5], and by the evidence that prolonged expiratory breathing promotes parasympathetic dominance as assessed by HRV [11,12], consistent with the autonomic improvements hypothesized to underlie the observed changes in AT-VO₂.

From an autonomic mechanistic standpoint, HRSB may approximate the individual's cardiovascular resonance frequency -- the breathing rate at which vagal baroreflex gain is maximized -- because the cardiac cycle period directly determines the respiratory cycle period in this paradigm. Lehrer et al. demonstrated that paced breathing at an individual's cardiovascular resonance frequency, delivered via HRV biofeedback, significantly increases vagal baroreflex gain and peak expiratory flow [9]. Enhanced baroreflex sensitivity would be expected to facilitate more efficient matching of cardiac output to metabolic demand during progressive exercise, potentially contributing to the observed increase in AT-VO₂. The role of cardiolocomotor synchronization described by Nomura et al. [1,2] and Bernasconi and Kohl, who demonstrated that intentional pacing of breathing to the exercise rhythm significantly increases the degree of locomotor-respiratory coordination [4], further supports the notion that rhythm entrainment during exercise can optimize oxygen kinetics. The broader applicability of externally paced breathing strategies during exercise has been further supported by Harbour et al., who demonstrated that evidence-based breathing techniques, including LRC, can reduce exercise-induced dyspnea and improve cardiorespiratory synchronization in runners [6,7].

The deterioration observed in Participant C is less straightforward to explain. Although this participant achieved a moderate entrainment rate (48%), consciously maintaining the 2:4 breathing ratio during incremental exercise may have imposed an excessive cognitive load, disrupting the automatic feedback regulation of ventilation. Under conditions of increasing metabolic demand, interference with chemoreceptor-driven ventilatory control could result in ventilatory inefficiency, reflected in the dramatic rise in VE/VO₂ (16.0→25.7) and the increase in perceived exertion. Matsumoto et al. noted that the effects of prolonged-expiration breathing on cardiopulmonary responses during incremental exercise vary with individual differences in autonomic nervous system responses [10], a finding consistent with their report of similar individual variation in autonomic responses to prolonged expiratory breathing at rest [11]. Participant C's outcome is consistent with these observations. It is also possible that the fixed 2:4 ratio was physiologically inappropriate for this individual, specifically, that it deviated substantially from his personal cardiovascular resonance frequency as identified through the biofeedback approach described by Lehrer et al. [9], resulting in autonomic dysregulation rather than optimization. Notably, it should also be acknowledged that all three participants showed similar entrainment rates (approximately 47%-49%), meaning that entrainment rate alone does not explain the divergent outcomes; the quality of cardiac-respiratory phase coupling and individual autonomic characteristics are likely more determinant factors.

These findings collectively suggest that HRSB is not uniformly beneficial and that individual assessment of resonance frequency and autonomic responsiveness may be a prerequisite for safe and effective implementation. Future studies should incorporate HRV frequency-domain analysis and muscle oxygen saturation (SmO₂) monitoring to elucidate the mechanistic pathways through which HRSB exerts its effects.

Several limitations of this study warrant acknowledgment. First, the sample comprised only three healthy young males, precluding statistical inference and limiting generalizability to females, older adults, and individuals with cardiorespiratory disease. Second, the fixed order of conditions (control on Day 1, intervention on Day 2) and the practice of HRSB between sessions introduce a potential order effect and expectation bias; future studies should employ randomized crossover designs with HRSB training provided before both conditions to minimize such bias. Third, each condition was measured only once per participant, without repeated measurements or calculation of the within-subject coefficient of variation, which reduces the reliability of the data and raises the possibility that observed changes reflect chance variation rather than a true intervention effect. Fourth, potential confounding factors such as caffeine intake, prior exercise, hunger status, and time of day were not fully controlled; although efforts were made to standardize testing conditions, these factors should be formally recorded and controlled in future studies. Fifth, the synchronization criterion (6 R-waves per respiratory cycle) was established post hoc; future protocols should define entrainment criteria a priori. Sixth, while the similar entrainment rates among participants (approximately 47%-49%) were used in part to discuss Participant C's divergent outcome, these rates are too similar across participants to serve as a meaningful explanatory variable for the observed differences; the discussion of this point has been revised accordingly.

In light of these limitations, several directions for future research are warranted. Replication in larger, statistically powered samples with repeated measurements is needed to confirm the present findings and to ensure the reliability of the data. Individualized optimization of the breathing ratio -- based on formal resonance-frequency assessment derived from resting HRV -- may help identify the most effective inspiratory-to-expiratory ratio for each participant and reduce the risk of adverse responses such as that observed in Participant C. Mechanistic investigation using HRV frequency-domain analysis and SmO₂ monitoring would further clarify the autonomic and peripheral circulatory pathways through which HRSB influences exercise tolerance. Finally, a randomized crossover design with adequate washout periods should be employed to eliminate order and expectation effects.

## Conclusions

This preliminary case series explored, for the first time, the potential influence of HRSB -- a breathing technique in which the respiratory cycle is entrained to the cardiac cycle at a 2:4 inspiratory-to-expiratory ratio -- on the AT during incremental exercise testing. Two of the three participants demonstrated improvements in AT-VO₂, VE/VO₂, and RPE under the HRSB condition, suggesting that HRSB may enhance ventilatory efficiency and exercise tolerance in some individuals through augmented parasympathetic activity and optimized baroreflex sensitivity. However, given the small sample size, single-measurement design, and fixed condition order, these findings should be interpreted with caution and regarded as preliminary observations rather than definitive evidence. The marked deterioration observed in one participant highlights the clinical importance of individual differences in autonomic response and respiratory control capacity, and underscores the need for individualized assessment before implementing HRSB in exercise or rehabilitation settings. Future controlled studies with larger samples, repeated measurements, and randomized designs are needed to confirm and extend these findings.
